# Water-Filtered Infrared A Irradiation in Combination with Visible Light Inhibits Acute Chlamydial Infection

**DOI:** 10.1371/journal.pone.0102239

**Published:** 2014-07-14

**Authors:** Hanna Marti, Maria Koschwanez, Theresa Pesch, Christian Blenn, Nicole Borel

**Affiliations:** 1 Institute of Veterinary Pathology, University of Zurich-Vetsuisse, Zurich, Switzerland; 2 Institute of Pharmacology and Toxicology, University of Zurich-Vetsuisse, Zurich, Switzerland; University of California, San Francisco, University of California, Berkeley, and the Children's Hospital Oakland Research Institute, United States of America

## Abstract

New therapeutic strategies are needed to overcome drawbacks in treatment of infections with intracellular bacteria. *Chlamydiaceae* are Gram-negative bacteria implicated in acute and chronic diseases such as abortion in animals and trachoma in humans. Water-filtered infrared A (wIRA) is short wavelength infrared radiation with a spectrum ranging from 780 to 1400 nm. In clinical settings, wIRA alone and in combination with visible light (VIS) has proven its efficacy in acute and chronic wound healing processes. This is the first study to demonstrate that wIRA irradiation combined with VIS (wIRA/VIS) diminishes recovery of infectious elementary bodies (EBs) of both intra- and extracellular *Chlamydia* (*C.*) in two different cell lines (Vero, HeLa) regardless of the chlamydial strain (*C. pecorum*, *C. trachomatis* serovar E) as shown by indirect immunofluorescence and titration by subpassage. Moreover, a single exposure to wIRA/VIS at 40 hours post infection (hpi) led to a significant reduction of *C. pecorum* inclusion frequency in Vero cells and *C. trachomatis* in HeLa cells, respectively. A triple dose of irradiation (24, 36, 40 hpi) during the course of *C. trachomatis* infection further reduced chlamydial inclusion frequency in HeLa cells without inducing the chlamydial persistence/stress response, as ascertained by electron microscopy. Irradiation of host cells (HeLa, Vero) neither affected cell viability nor induced any molecular markers of cytotoxicity as investigated by Alamar blue assay and Western blot analysis. Chlamydial infection, irradiation, and the combination of both showed a similar release pattern of a subset of pro-inflammatory cytokines (MIF/GIF, Serpin E1, RANTES, IL-6, IL-8) and chemokines (IL-16, IP-10, ENA-78, MIG, MIP-1α/β) from host cells. Initial investigation into the mechanism indicated possible thermal effects on *Chlamydia* due to irradiation. In summary, we demonstrate a non-chemical reduction of chlamydial infection using the combination of water-filtered infrared A and visible light.

## Introduction

The *Chlamydiaceae* are implicated in a wide variety of acute and chronic diseases in both animals and humans [1]. Trachoma induced by *Chlamydia (C.) trachomatis* is the leading cause of preventable blindness of human beings in developing countries. Other *C. trachomatis* serovars are the principal bacterial cause for sexually transmitted diseases leading to sterility in women in developed countries [2]. *C. pecorum* is associated with various diseases ranging from keratoconjunctivits to pneumonia, polyarthritis and abortion in swine and ruminants [3]. Unlike most bacterial species, chlamydiae replicate within a membrane-bound vacuole termed an inclusion in the cytoplasm of host cells. The obligate intracellular lifestyle of chlamydiae is characterized by a biphasic developmental cycle consisting of an infectious and metabolically less active stage (elementary body or EB), which differentiates into dividing reticulate bodies (RBs) before transforming back into EBs [4]. Several factors such as exposing acutely infected cells to IFN-γ can induce the persistence/stressed state, which may enable chlamydiae to withstand hostile conditions within the cell. Persistence is defined as a viable but non-infectious developmental state that is: i) reversible once the stressor is removed; and ii) characterized by the presence of aberrant bodies (ABs), which show distinct morphological differences compared to EBs or RBs. Other persistence inducers include deprivation of glucose, iron or amino acids, and heat shock (reviewed in [5]).

The complex lifestyle of *Chlamydiaceae* poses challenges to both diagnosis and treatment of chlamydial infections. Antimicrobial therapy is the treatment of choice for bacterial infections. However, in *Chlamydia*, concerns about the possible development of resistance during antibiotic therapy have discouraged the use of certain antimicrobial compounds for chlamydial treatment (i.e. rifampin resistance in *C. trachomatis* [6]–[8]). Furthermore, there is evidence that β-lactam antibiotics induce persistence *in vitro* as well as *in vivo* (reviewed in [9]). Another concern is insufficient compliance regarding multiple-dose treatment over the course of several days (reviewed in [10]). New therapeutic strategies are therefore needed to overcome drawbacks in treatment of chlamydial infections.

Light of a halogen bulb passing a water-containing cuvette emits water-filtered infrared A (wIRA) and visible light (VIS) as previously shown [11]. WIRA is short wavelength infrared radiation with a spectrum ranging from 780 to 1400 nm. WIRA alone and in combination with VIS (wIRA/VIS) has been used in different clinical settings and its efficacy has been proven in acute and chronic wound healing processes [12]. Only few preliminary data on treatment of infectious conditions with wIRA irradiation have been reported so far. A lower rate of wound infections following abdominal surgery was observed after post-operative irradiation with wIRA/VIS compared to treatment with VIS [11]. However, its direct effect on pathogens *in vitro* and in particular on obligate intracellular infectious agents such as chlamydiae has not been shown before.

The aim of this study was to investigate the effect of wIRA/VIS on *Chlamydia*, on two types of mammalian host cells and on their interaction in a cell culture model. Here we demonstrate that EBs are less infectious after wIRA/VIS treatment compared to non-treated control EBs regardless of the chlamydial strain. Irradiation of cells did not induce cytotoxicity even at high wIRA/VIS doses and long-time exposure. Furthermore, it could be shown that single-dose irradiation caused a significant reduction of chlamydial inclusions without inducing persistence. Three-fold irradiation led to an even more profound reduction. Irradiation and chlamydial infection triggered an identical host cell response measured by the release of a cluster of cytokines and chemokines. Finally, heating experiments resulted in a reduction of the chlamydial burden comparable to the wIRA/VIS treatment indicating a potential thermal-induced mode of action.

## Materials and Methods

### Host cells and media

Vero 76 cells (African green monkey kidney cells, CRL 1587 American Type Culture Collection (ATCC), Manassas, VA, USA) and HeLa cells (Homo sapiens cervix adenocarcinoma, CCL-2 ATCC) were cultured at 37°C with 5% CO_2_ in growth culture medium for cell propagation. Vero growth medium consisted of Minimal Essential Medium (MEM) with Earle's salts, 25 mM HEPES, without L-Glutamine (GIBCO, Invitrogen, Carlsbad, CA, USA) supplemented with 10% fetal calf serum (FCS, BioConcept, Allschwil, Switzerland), 4 mM GlutaMAX-I (200 mM, GIBCO) and 0.2 mg/ml gentamycin (50 mg/ml, GIBCO). HeLa cell culture media were further supplemented with 1% MEM Non-Essential Amino Acids (MEM NEAA, 100x, GIBCO). Medium used for cell propagation intended for infection experiments was without gentamycin [13]. Cells were seeded on round glass coverslips (13 mm diameter, Sterilin Limited (Thermo Fisher Scientific), Cambridge, UK) in 24-well plates (Techno Plastic Products AG (TPP), Trasadingen, Switzerland) at a density of 3×10^5^/well in 1 mL medium for infection experiments. Infection experiments were performed when cells reached at least 90% confluency.

### Chlamydial strains

In this study, two different strains of *Chlamydiaceae* were used for *in vitro* infection experiments: *Chlamydia (C.) pecorum* 1710S (isolate from a swine abortion, kindly provided by Prof. J. Storz, Baton Rouge, LA, USA) and *C. trachomatis* serovar E (kindly provided by Prof. R. V. Schoborg, Johnson City, TN, USA). The isolate of the *C. trachomatis* strain was originally obtained from S. P. Wang and C.-C. Kuo (University of Washington, Seattle, WA, USA). Subsequently, it was propagated and harvested as previously described [14]. Briefly, *C. trachomatis* stocks were propagated in HEC-1B cells, supplemented with 2 mM glutamine and 10% FCS for 72 h, harvested in 2-fold sucrose-phosphate-glutamate (SPG) and stored at −80°C. Stocks of *C. pecorum* were propagated in HEp-2 monolayers, purified and stored at −80°C in SPG medium as shown previously [13].

### wIRA irradiation

If not stated differently, cultures were exposed to water-filtered infrared A combined with visible light (wIRA/VIS) for 20 min using a wIRA radiator (hydrosun 750, Hydrosun GmbH, Müllheim, Germany) at a dose of 3700 W/m^2^. The resulting radiation spectrum ranges from 380 nm up to 1400 nm. The 24-well plate was placed in a thermostat-controlled water bath (SC100, Thermo Fisher Scientific, Newington, CT, USA), which maintained a temperature of 37°C and was used as a cooling system for the irradiated cultures as previously described [15]. Non-irradiated controls were localized on the same plate and were kept at a suitable distance from the irradiated wells in order to avoid any direct and/or indirect irradiation influence (data not shown). Additionally, the temperature was monitored using a Voltcraft thermometer (Type 2ABAc, Philips, Kassel, Germany).

### Study design

#### Infection Experiments

The experiments were organized in four different treatment groups: i) non-infected cells without irradiation, ii) non-infected cells with irradiation, iii) *Chlamydia*-infected cells without, and iv) *Chlamydia*-infected cells with irradiation. Vero and HeLa cells were infected with either *C. pecorum* or *C. trachomatis* at 1 multiplicity of infection (MOI) in 1 mL infection medium (growth culture medium without FCS and gentamycin). After centrifugation for 1 hour (h) at 1000 g and 25°C, infection medium was replaced by incubation medium (growth culture medium without gentamycin) containing 1% cycloheximide (Sigma-Aldrich, Inc., St. Louis, MO, USA) and cultures were incubated at 37°C as previously described [13].

#### Irradiation of chlamydial EBs prior to host cell infection

Chlamydial EBs at MOI 1 were irradiated in 1 mL infection medium for 20 min at a dose of 3700 W/m^2^ before infection of host cells. Non-irradiated EBs were used as controls. After an incubation period of 43 h, cultures were subjected to i) immunofluorescence microscopy, or ii) infectious titer analysis, as appropriate. *C. pecorum* EBs were used to infect Vero and HeLa monolayers. *C. trachomatis* EBs were applied to HeLa cells only.

#### Single-dose irradiation 40 hours post infection

**Figure 1:**
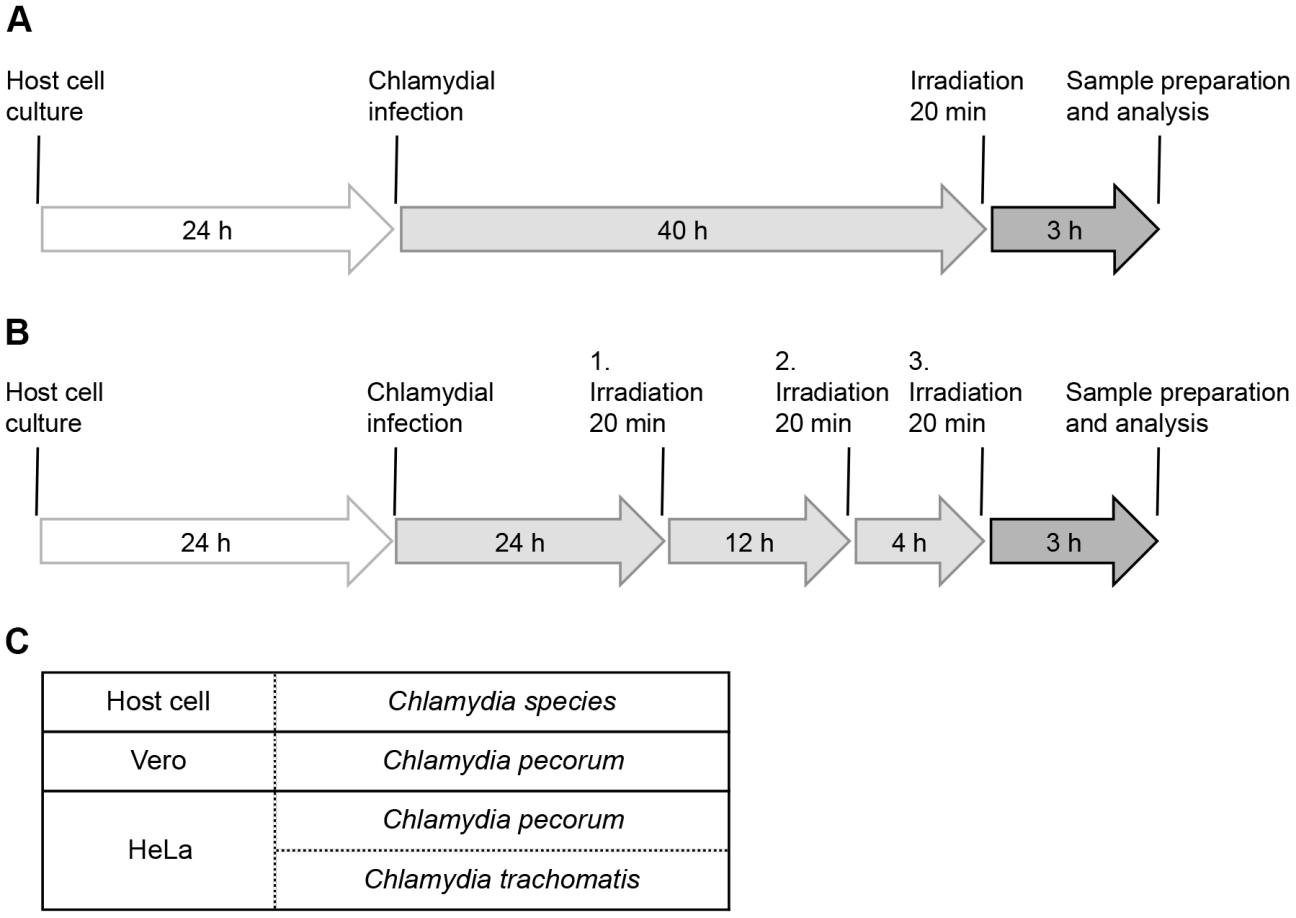
Study Design. (A) Diagram of the infection and single-dose irradiation procedure. Host cells were infected, incubated for 40 h and irradiated for 20 min. After another incubation period of 3 h, samples were further processed and analyzed. (B) Triple-dose irradiation: general design is according to A with additional irradiations (20 min) at 24 and 36 hours post infection (hpi). (C) Table shows host cells and chlamydial strains investigated within this study.

For single-dose irradiation, HeLa and Vero monolayers were infected with *C. trachomatis* or *C. pecorum*. After an incubation period of 40 h at 37°C, cultured cells were irradiated for 20 min (3700 W/m^2^). Cultures were incubated for another 3 h at 37°C under standard culture conditions before being subjected to i) immunofluorescence microscopy, ii) infectious titer analysis, or (iii) transmission electron microscopy (TEM) (Figure 1A) if not stated differently. Vero monolayers were infected with *C. pecorum* and HeLa cells were infected with *C. trachomatis*.

#### Triple-dose irradiation 24, 36 and 40 hours post infection

For multiple-dose irradiation, *Chlamydia*-infected cultures were incubated for 24 h at 37°C. Irradiation was performed three times, at 24 hours post infection (hpi), 36 hpi and 40 hpi for 20 min with the above mentioned standard dose. At 43 hpi, cultures were further processed for i) immunofluorescence microscopy, ii) infectious titer analysis, iii) TEM and iv) cytokine and chemokine assays (Figure 1B). HeLa cells were infected with *C. trachomatis* (Figure 1C).

### Cell viability assays

10% Alamar blue dye was added to non-infected cell cultures at specified times following irradiation. After 3 h of incubation at 37°C, fluorescence was monitored at 530-nm excitation and 590-nm emission wavelength (LS55 luminescence spectrometer, Perkin-Elmer) as previously described [16] Dosage and duration of irradiation depended on the experiment.

### Western blot analysis

Western blot experiments were performed to assess molecular markers of cytotoxicity in irradiated HeLa cells. Non-infected HeLa monolayers were irradiated for 20 min (3700 W/m^2^) and harvested directly into Laemmli buffer at specific time points depending on the experiment. Samples were further processed and analyzed as described previously [17]. Proteins were detected using the following primary antibodies: anti-AKT (11E7, 1∶1000; Cell Signaling, Danvers, MA, USA), anti-pAKT (S473, 1∶4000; R&D Systems, Minneapolis, MN, USA), anti-p38 mitogen-activated protein kinase (MAPK, 1∶1000; Cell Signaling), anti-phospho-p38 MAPK (Thr180/Tyr182, 1∶1000; Cell Signaling), anti-ERK1/2 (1∶1000; R&D Systems), anti-phospho-p44/42 MAPK (ERK1/2, Thr202/Tyr204, 1∶1000; Cell Signaling), anti-actin (clone 4, 1∶5000; Millipore, Billerica, MA, USA), anti-caspase-7 (cleaved, Asp198, 1∶1000; Cell Signaling), anti-caspase-9 (cleaved, Asp330, 1∶1000; Cell Signaling), anti-LCA/B (G40, 1∶1000; Cell Signaling). All secondary antibodies were purchased from Sigma. Signals were detected by enhanced chemiluminescence (ECL; ECL detection kit, Pierce, Rockford, IL, USA). Samples of H_2_O_2_ treated HeLa cells were analyzed in parallel as positive Western blot controls.

### Immunofluorescence microscopy

Monolayers were fixed with absolute methanol (−20°C) for 10 min and immunolabelled as described [13]. Briefly, chlamydial inclusions were detected using a *Chlamydiaceae* family-specific mouse monoclonal antibody directed against the chlamydial lipopolysaccharide (LPS, Clone ACI-P, 1∶200; Progen, Heidelberg, Germany) and a 1∶500 diluted Alexa Fluor 488-conjugated secondary goat anti-mouse antibody (Molecular Probes, Eugene, OR, USA). Host and chlamydial DNA were labeled using 1∶1000 diluted 4′, 6-Diamidin-2′-phenylindoldihydrochlorid (DAPI, Molecular Probes). Coverslips were mounted with FluoreGuard Mounting (Hard Set, ScyTek Laboratories Inc., Logan, UT, USA) on glass slides and assessed using a Leica DMLB fluorescence microscope (Leica Microsystems, Wetzlar, Germany) under oil immersion at 1000-fold magnification with a 100× objective (PL FLUOTAR 100x/1.30, OIL, ∞/0.17/D, Leica Microsystems) and a 10× ocular objective (Leica L-Plan 10x/25 M, Leica Microsystems). In total, 200 cell nuclei and all chlamydial inclusions per examined field were counted. Microscopic images were captured with the BonTec measuring and archiving software (BonTec, Bonn, Germany) using a UI-2250SE-C-HQ camera (uEye, IDS Imaging Development Systems GmbH, Obersulm, Germany).

### Chlamydial titration by sub-passage

Depending on the experiment, monolayers were scraped at 43 hpi and stored at −80°C, either separately or together with supernatant, followed by sub-passage on the respective host cell as previously described [13] with minor modifications: centrifugation was omitted and the suspension consisting of both cells and supernatant was sonificated for 5 min (Branson Sonifier 250; Branson Ultrasonics, Danbury, CT, USA) before reinfection experiments. Fixation and immunofluorescence staining was performed as described above. The number of inclusions in 30 random microscopic fields per sample was determined using a Leica fluorescence microscope at 200-fold magnification with a 20× objective (PL FLUOTAR 20x/0.50 PH 2, ∞/0.17/B) and a 10× ocular objective (Leica L-Plan 10x/25 M, Leica Microsystems). The number of inclusion-forming units (IFU) in undiluted inoculum was then calculated and expressed as IFU per ml inoculum according to previously published methods [18].

### Transmission electron microscopy (TEM)

HeLa and Vero monolayers were fixed at 43 hpi in 2.5% gultaraldehyde (Electron Microscopy Sciences, Ft. Washington, USA) for 1 h and embedded in epoxy resin (Fluka) by routine methods. Further preparation and investigation were performed as described previously [13]. Ultrathin sections (80 nm) were mounted on gold grids (Merck Eurolab AG, Dietlikon, Switzeland), contrasted with uranyl acetate dehydrate (Fluka) and lead citrate (Merck Eurolab AG). The sections were investigated in a Philips CM10 electron microscope. The total number of bacteria in ten inclusions per condition was counted. Additionally, chlamydial bacteria were classified according to their morphology into EBs (dark, 0.25–0.5 µm), IBs (dark center and pale periphery), RBs (pale, 0.5–1 µm) and ABs (pale, ≥2 µm).

### Cytokine and chemokine assay

Cytokine and chemokine assays were performed three times to investigate the host cell response to irradiation, chlamydial infection and the combination of both, respectively. Following threefold irradiation, 1 mL of supernatant per well was collected 43 hpi and stored at −80°C until further processing. One supernatant of each experimental group was thawed and then filtered by using a 0.2 µm syringe filter (Whatman FP 13/0.2 RC-2, Sigma-Aldrich) followed by filtration through a 0.1 µm syringe filter (Whatman Anotop 10, Sigma-Aldrich) to remove all infectious EBs according to published methods [19]. Cytokines and chemokines were measured using ProteomeProfiler antibody arrays (Human Cytokine Array Panel A kit and Human Chemokine Array Kit, R&D Systems, Minneapolis, MN, USA). 500 µ of filtered supernatant per condition was processed according to manufacturer's instructions. Signals were detected by ECL (ECL detection kit, Pierce, Rockford, IL, USA) and analyzed using Adobe Photoshop CS6. First, the arrays were validated by evaluating the linearity of the internal assay controls determined over time for each condition (Figure S2). The mean pixel density of the positive controls was measured over time using the Adobe Photoshop histogram tool [20]. The area to be measured was defined as 28×28 pixels encompassing the entire positive control spot. Subsequently, the mean pixel density of each spot representing cytokines, chemokines, positive and negative controls was analyzed after three minutes of exposure to ECL. The pixel densities of six spots per positive control were averaged and set to 100%. Two spots per cytokine or chemokine were averaged and expressed as percentage of the positive control according to published methods [21].

### Statistical analysis

All statistical analyses were performed with PRISM software (GraphPad, Version 5.0b). If not stated differently, all results are displayed as means ± standard deviation (SD) or as means ± standard error of the mean (SEM) of the indicated number of experiments. The significance of differences was estimated by *t* test, and a p-value of <0.05 was considered significant.

## Results

### Irradiation of chlamydial EBs reduces their infectivity on host cells

**Figure 2:**
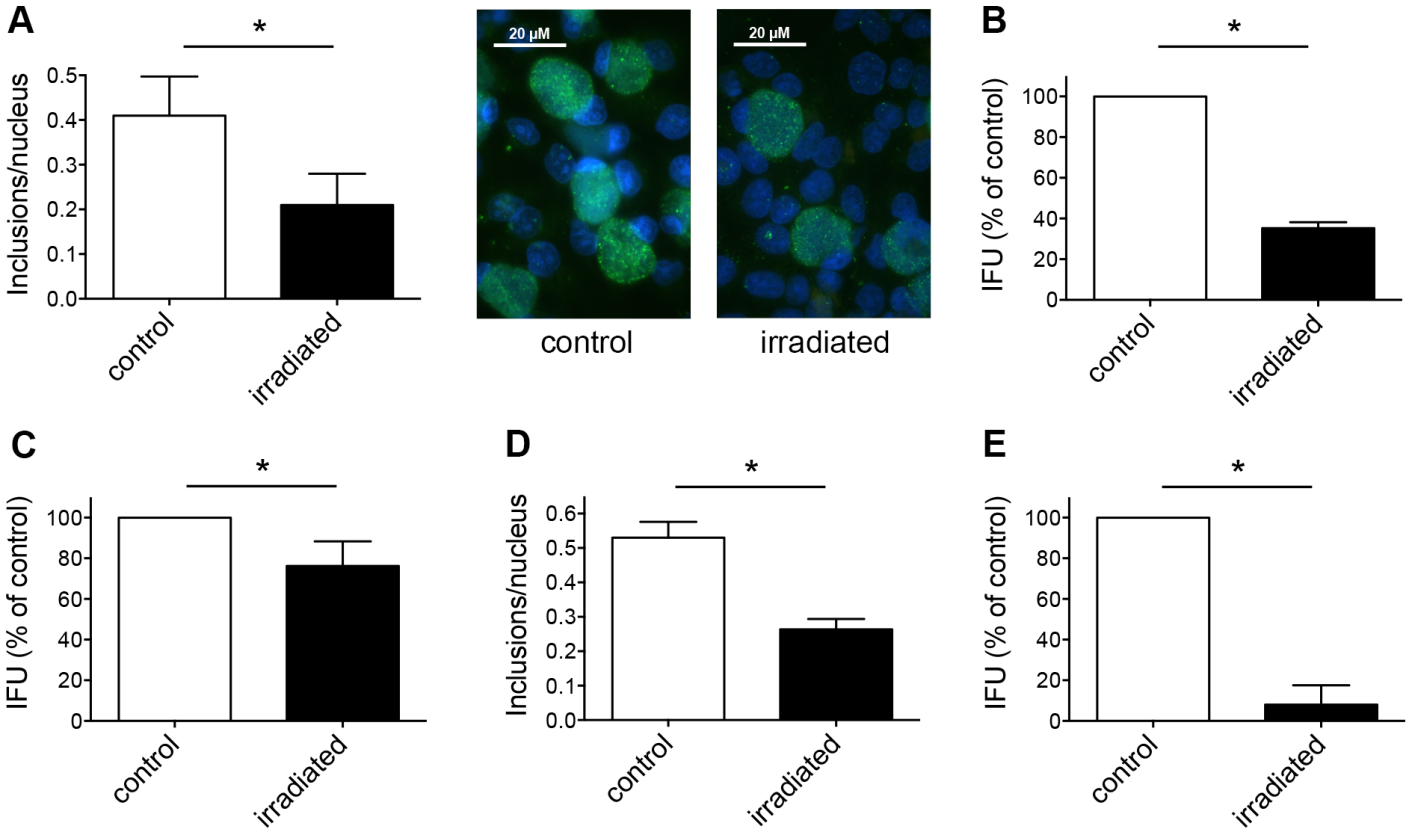
Irradiation of chlamydial EBs reduces their infectivity on host cells. (A) *C. trachomatis* EBs (MOI 1) were either irradiated or not prior to infection of HeLa monolayers. Cultures were incubated for 43 hours, fixed, and immunolabelled with anti-chlamydial LPS (green) and DAPI (blue). Frequency of inclusions per nucleus was calculated (mean ± SD; * p<0.05; n = 3; *t* test). Representative microscopic pictures at 1000× magnification are shown additionally in the panel. (B) Cultures were collected and subjected to sub-passage titer analysis. Inclusion forming units (IFU) are shown as percent of control (mean ± SD; * p<0.0001; n = 3; *t* test). (C) *C. pecorum* EBs were irradiated prior to infection of HeLa monolayers. Non-irradiated EBs were used as controls. Cultures were collected and subjected to sub-passage titer analysis. Inclusion forming units (IFU) are presented as percent of control (mean ± SD; * p<0.05; n = 3; *t* test). (D) *C. pecorum* EBs were irradiated or not prior to infection of Vero monolayers. Cultures were incubated for 43 hours, fixed, and immunolabelled as shown in panel A. Frequency of inclusions per nucleus was calculated (mean ± SD; * p<0.0025; n = 3; *t* test). (E) Cultures from panel D were collected and subjected to sub-passage titer analysis. Inclusion forming units (IFU) are shown as percent of control (mean ± SD; * p<0.0001; n = 3; *t* test).

To determine the effect of irradiation on *Chlamydia* alone, we irradiated EBs of two different chlamydial strains prior to infection of host cells. First, we infected HeLa monolayers with irradiated *C. trachomatis* EBs whereas non-irradiated EBs were used as controls. Cultures were incubated for 43 h and either collected for sub-passage analysis, or fixed and immunolabelled directly. The infectivity rate of irradiated EBs was lower (0.21±0.07 inclusions per nucleus) than that of the controls (0.41±0.09 inclusions/nucleus) (Figure 2A). These findings were further confirmed by sub-passage analysis. IFU/mL was calculated and expressed as percentage of the control (34.72%±3.24%, Figure 2B), showing differences between the treated and non-treated groups. In order to evaluate whether this effect was limited to a specific chlamydial strain, we infected HeLa monolayers with irradiated *C. pecorum* EBs using a similar setting. IFU/mL of irradiated EBs was lower after titration by sub-passage (76.2%±12.11%) compared to 100% of the non-irradiated control (Figure 2C). Furthermore, in order to exclude that the effect was host cell-related, we used irradiated *C. pecorum* EBs to infect Vero cells. The frequency of *C. pecorum* chlamydial inclusions per Vero cell nucleus was reduced if EBs were irradiated prior to infection (0.26±0.03 inclusions/nucleus) compared to non-irradiated *C. pecorum* control EBs (0.53±0.05 inclusions/nucleus, Figure 2D). Subsequent titration by sub-passage led to a more pronounced reduction of *C. pecorum* EBs in Vero cells (8.0%±9.52% compared to 100% of the control, Figure 2E) than irradiation of *C. trachomatis* EBs prior to infection of HeLa cells.

### Irradiation of mammalian cells does not induce general and molecular markers of cytotoxicity

**Figure 3:**
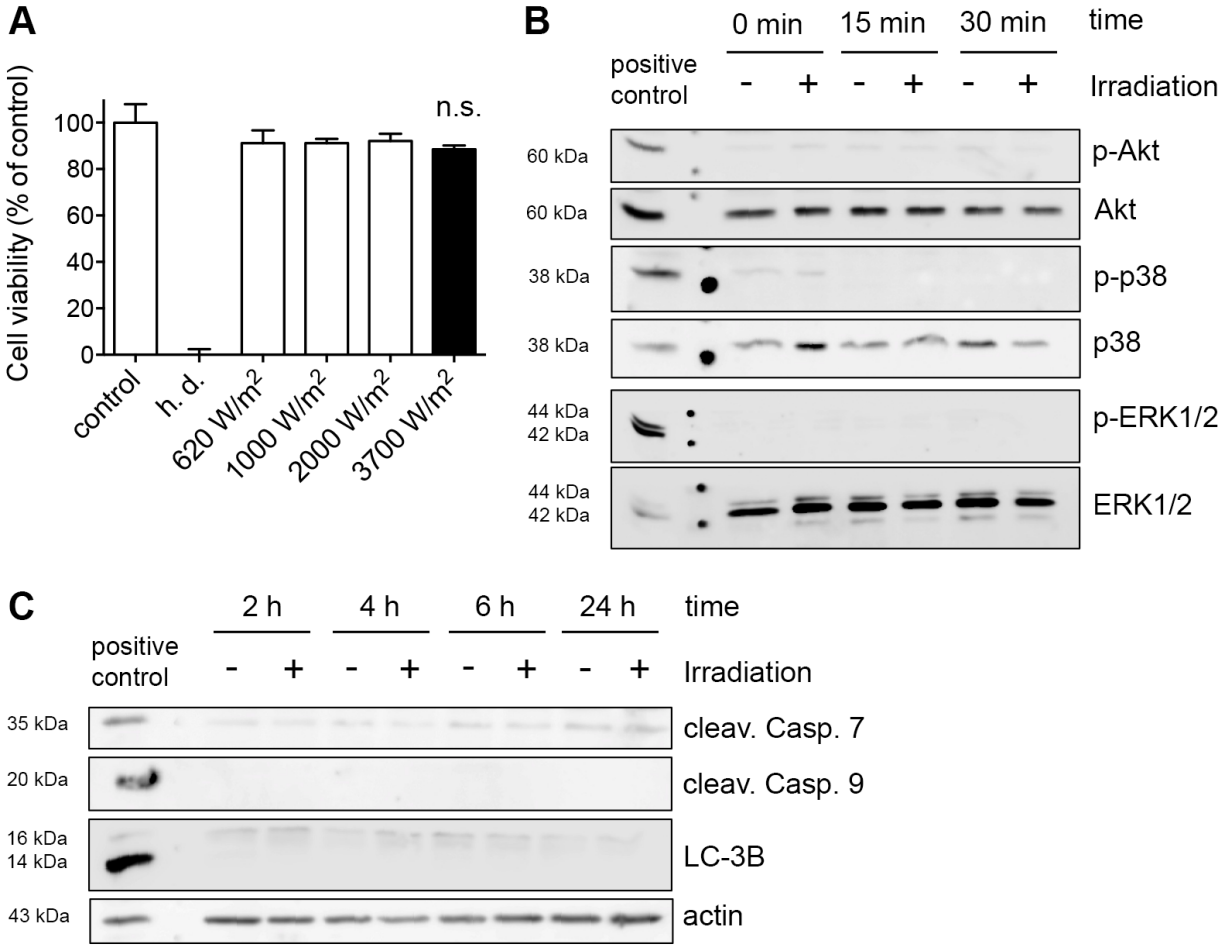
Irradiation of mammalian cells does not induce general and molecular markers of cytotoxicity. (A) Vero cells were irradiated with the indicated dosage for 20 min. Cell viability was analyzed using Alamar blue dye 3 h after irradiation (mean ± SD; n.s. non significant; n≥3; *t* test). Heat-denaturated samples (h.d.) were included as killing controls. (B) HeLa monolayers were irradiated for 20 min with 3700 W/m^2^ and subsequently collected at the indicated time points. Western blot analysis of phosphorylated AKT (pAKT), p38 (p-p38) and ERK 1/2 (p-ERK1/2) are shown. Unphosphorylated proteins are presented as loading controls. (C) Western blot analysis of cleaved Caspase 7 and 9 as well as LC-3B in HeLa cells after irradiation (20 min) at the indicated time points. Actin was used as loading control.

Next, we evaluated the impact of irradiation dose and duration on two different cell lines (Vero, HeLa) to investigate potential cytotoxic side effects due to irradiation. First, cell viability was tested by the Alamar blue assay in the Vero cell line. In these experiments, we irradiated non-infected Vero cells for 20 min at doses ranging from 620 to 3700 W/m^2^, respectively, and incubated them for 3 h before adding the Alamar blue dye. After another incubation period of 3 h, we monitored the fluorescence to quantify cell viability. Heat-denaturated (h.d.) Vero cells were included within the experiments as controls. The overall cell viability was not affected by irradiation regardless of the exposure intensity, even at the highest achievable dose of 3700 W/m^2^ (Figure 3A). Then we investigated irradiated HeLa cells using the Alamar blue assay. Irradiation was applied at a dose of 3700 W/m^2^ for 20 min as above and irradiation time was extended to 60 and 240 min. Cell viability was assessed 0, 30 min, 6 h and 24 h after treatment and compared to non-irradiated controls. No significant decrease of cell viability in any of these settings was observed (Figures S1A, S1B and S1C).

To gain more information towards possible cell stress signaling due to irradiation, well-known molecular markers of cytotoxicity were evaluated in irradiated HeLa cells by Western blot analysis [22]. HeLa cells exposed to irradiation for 20 min at a dose of 3700 W/m^2^ were investigated for the presence of phosphorylated forms of the stress kinases Akt, p38 and ERK 1/2, at 0, 15 and 30 min after irradiation. We observed no difference between irradiated and non-irradiated cells regarding the phosphorylation status of Akt, p38 and ERK 1/2 protein (Figure 3B). Samples were also collected 2, 4, 6 and 24 h after treatment and examined for autophagy (LC-3B) and apoptosis induction (cleaved Caspases 7 and 9). Irradiation did not induce either apoptosis or autophagy marker regardless time post-irradiation in HeLa cells (Figure 3C).

### A single dose of irradiation reduces the number of chlamydial inclusions within host cells

**Figure 4:**
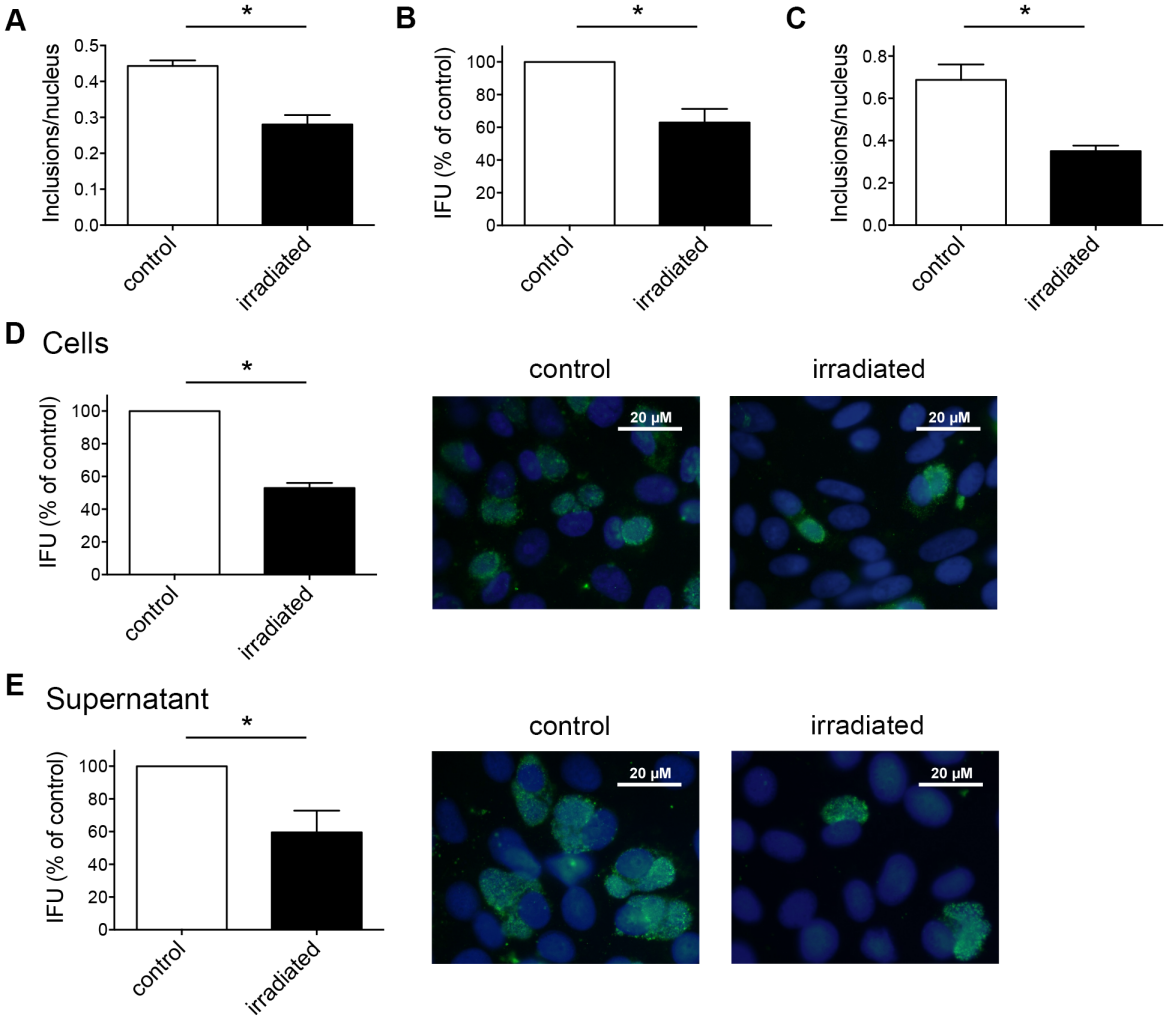
A single dose of irradiation reduces the number of chlamydial inclusions within host cells. (A) *C. trachomatis*-infected HeLa cells were either irradiated or not 40 hpi. Cultures were fixed 43 hpi and immunolabelled with anti-chlamydial LPS (green) and DAPI (blue). Frequency of inclusions per nucleus was calculated (mean ± SD; * p<0.001; n = 3; *t* test). (B) Infected HeLa cultures were collected and subjected to sub-passage titer analysis. Inclusion forming units (IFU) are shown as percent of control (mean ± SD; * p<0.0025; n = 3; *t* test). (C) Vero monolayers infected with *C. pecorum* were irradiated 40 hpi. Non-irradiated, *C. pecorum*-infected cells were used as controls. Cultures were incubated for 46 hours, fixed, and processed as in panel A (mean ± SD; * p<0.0025; n = 3; *t* test). (D) *C. pecorum*-infected Vero cells were separated from the supernatant, collected 43 hpi and subjected to sub-passage titer analysis. Monolayers were fixed 40 h post reinfection and immunolabelled as shown in panel A. Inclusion forming units are shown as percent of control (mean ± SD; * p<0.0001; n = 3; *t* test). Representative microscopic pictures at 1000× magnification are shown additionally in the panel. (E) The supernatant of the infected cells (panel D) was collected separately and subjected to sub-passage titer analysis. Inclusion forming units are represented as percent of control (mean ± SD; * p<0.01; n = 3; *t* test). Representative pictures are presented as well.

After cytotoxic effects on host cells due to irradiation were precluded, we investigated the effect of a single dose of irradiation applied to infected cell monolayers. We aimed to determine whether irradiation has an effect on mature inclusions containing EBs and RBs within the host cell. We infected HeLa monolayers with *C. trachomatis* and irradiated them at 40 hpi for 20 min at a dose of 3700 W/m^2^. Parallel non-irradiated but infected HeLa cultures were used as controls. Similar to previous experiments, cultures were either collected for sub-passage titer analysis, or fixed and directly immunolabelled. Again, the frequency of chlamydial inclusions per nucleus was reduced in irradiated cells (0.28±0.02 inclusions/nucleus) compared to non-irradiated *C. trachomatis*-infected HeLa cells (0.44±0.02) (Figure 4A). These findings were confirmed by sub-passage titer analysis showing that the IFU/mL of irradiated cultures was 62.88%±8.41% compared to the control (Figure 4B). The reduction of inclusions per nucleus in Vero cells infected with *C. pecorum* was confirmed in parallel experiments and was similar (0.35±0.03 inclusions/nucleus compared to the control with 0.69±0.07) (Figure 4C).

It was possible that irradiation of *Chlamydia*-infected host cells induces premature rupture of intracytoplasmic chlamydial inclusions, leading to loss of intact inclusions and subsequent release of EBs into the supernatant. Therefore, we assessed shedding of EBs into the supernatant by collecting the supernatant and the infected cell monolayer as separate fractions and analyzing them individually by sub-passage titer analysis. IFU was similarly reduced up to 50% by irradiation in both cells and supernatant (52.94%±3.13% reduction in monolayers (Figure 4D) and 59.58%±13.27% in supernatants (Figure 4E)).

**Figure 5:**
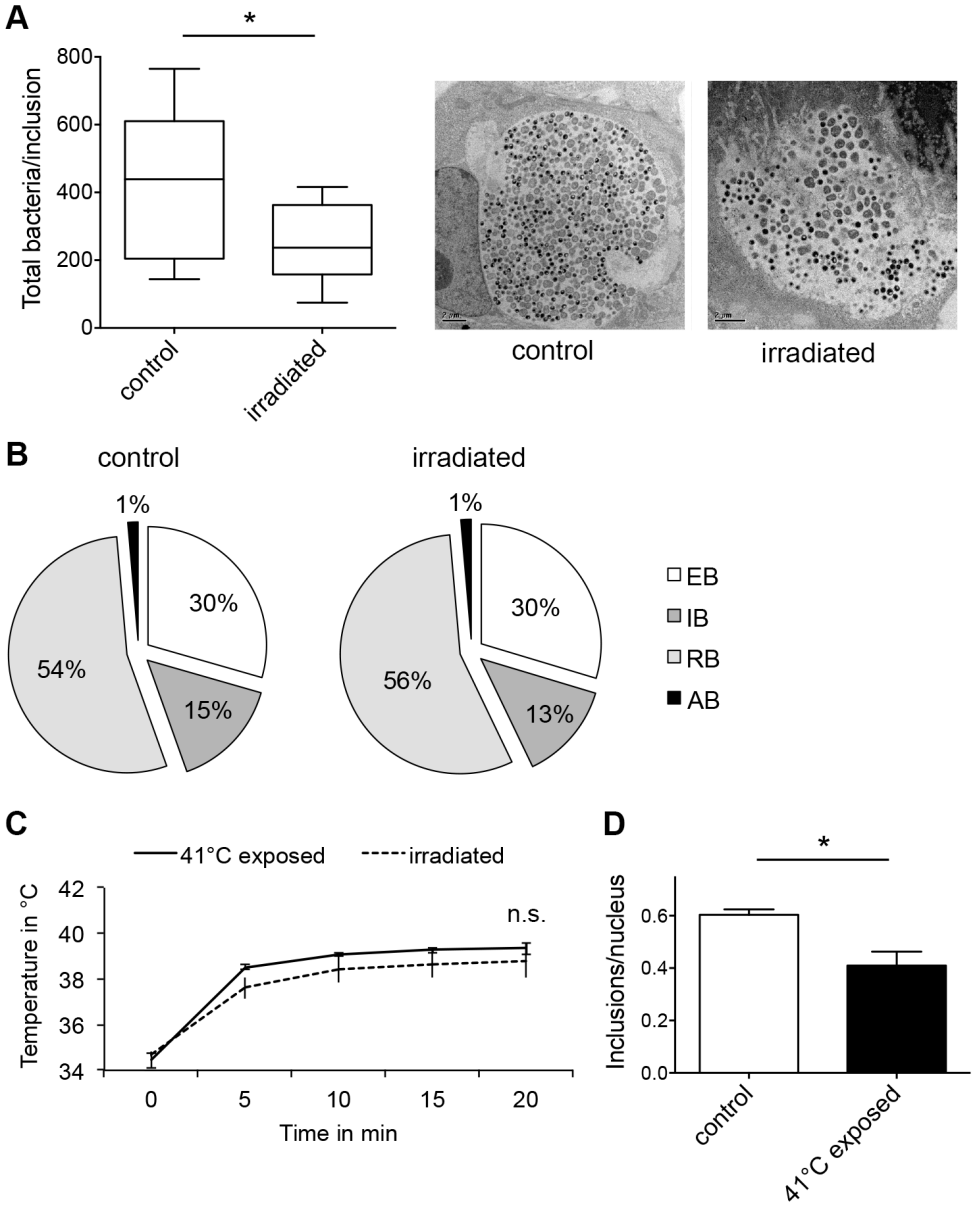
Thermal effects of irradiation are responsible for the reduced chlamydial infectivity. (A) *C. pecorum*-infected Vero cells were irradiated 40 hpi. Non-irradiated, *C. pecorum*-infected cells are controls. Cultures were fixed 43 hpi with glutaraldehyde, and further processed as described in Material and Methods in the transmission electron microscopy section. The total number of bacteria in ten inclusions per condition was counted: controls (in sum 4318 bacteria) and irradiated inclusions (2524 bacteria). Shown are the means ± SD of ten chlamydial inclusions (* p<0.05, n = 10; *t* test). The mid-line shows the median, the box represents the 25^th^ to 75^th^ percentiles whereas the whiskers show the minimum to maximum values. Representative inclusions are shown additionally in the panel. (B) Chlamydial bacteria within ten inclusions (from A) were split according to their morphology into elementary bodies (EB), intermediate bodies (IB), reticulate bodies (RB) and aberrant bodies (AB). The graphs show the distribution of each maturation stage per condition. (C) 1 mL of cell culture medium in a well of a 24-well plate was irradiated or the resulting temperature enhancement due to irradiation was mimicked using a water bath at 41°C (41°C exposed). Shown is the intra-well temperature at the indicated time points (mean ± SD; n.s. non significant, n = 3). (D) Vero cells were infected with *C. pecorum*, incubated for 40 h and placed in a water bath at 41°C for 20 min (41°C exposed). In parallel, *C. pecorum*-infected Vero cultures were placed in a water bath at 37°C for 20 min and were used as controls. Cultures were subsequently fixed at 43 hpi and immunolabelled with anti-chlamydial LPS (green) and DAPI (blue). Frequency of inclusions per nucleus was calculated (mean ± SD; * p<0.005; n = 3; *t* test).

Irradiation of chlamydial inclusions at 40 hpi might also reduce chlamydial infectivity by influencing chlamydial maturation or inducing persistence/stress, which is characterized by alteration of the chlamydial developmental cycle and the presence of aberrant bodies (ABs). Therefore, the ultrastructure of irradiated chlamydial inclusions at 43 hpi was evaluated and compared to that of the infected but non-irradiated controls by transmission electron microscopy (TEM). Vero cells were infected with *C. pecorum*, irradiated and processed as described before. First, ten inclusions for each condition (irradiated inclusions, non-irradiated inclusions) were picked and the total number of bacteria was counted and allocated to the different developmental stages (EBs, RBs, IBs, ABs) according to their morphology. With a total of 4318 chlamydial bodies, inclusions within non-irradiated samples contained almost as twice as many bacteria as the irradiated inclusions (2524 chlamydial bodies, Figure 5A). There was no difference in the distribution of the different chlamydial maturation stages between irradiated and non-irradiated chlamydial inclusions. The irradiated inclusions harbored 30%±8.85% EBs, 13.4%±3.89% IBs, 56.4%±11.02% RBs and 1.4% ABs, whereas the non-irradiated chlamydial inclusions consisted of 29.8%±9.91% EBs, 15.3%±3.83% IBs, 54.7%±10.92% RBs and 1.4% ABs (Figures 5B and S3A).

### Thermal effects of irradiation are responsible for the reduced chlamydial infectivity

To elucidate the basal mechanism of chlamydial reduction due to irradiation, we measured the intra-well temperature on top of the coverslip at the bottom of the well during the 20 min irradiation period at the applied dose of 3700 W/m^2^. The temperature profile started with an initial rise of temperature from 34.7°C up to 37.6°C during the first 5 min of irradiation. After ten minutes, the intra-well temperature reached a stationary phase at 38.4°C and did not exceed 38.8°C within the 20 min irradiation period. Non-irradiated wells on the same plate were used as controls. The temperature at the bottom of the control-wells varied between 35.1°C and 35.9°C within the 20 min time frame. The temperature raise induced by the irradiation was then mimicked by heating the water bath to 41°C for 20 min showing identical temperature kinetics (Figure 5C).

Subsequently, the setting of single-dose irradiation was mimicked by placing *C. pecorum*-infected Vero cells (40 hpi) in a water bath at 41°C for 20 min without any irradiation. Cultures were fixed and immunolabelled at 43 hpi as previously described. In parallel, *C. pecorum*-infected Vero cultures were kept in a water bath for 20 min at 37°C (untreated controls). On the same plate, separate wells containing *C. pecorum*-infected Vero cells were irradiated with wIRA/VIS for 20 min (3700 W/m^2^). The frequency of chlamydial inclusions was reduced in the temperature-treated cultures (0.41±0.05 inclusions/nucleus) compared to that of untreated controls (0.61±0.02 inclusions/nucleus) (Figure 5D) similarly to the effect of single-dose irradiation (0.47±0.03 inclusions/nucleus).

The potential cytotoxic effect of the temperature treatment was evaluated in HeLa cells by testing the cell viability with the Alamar blue. Non-infected HeLa monolayers were placed in a water bath at 41°C for 20 min and Alamar blue was added at 0, 30 min, 6 and 24 h after the treatment. We found no decrease of cell viability at any of the analyzed time points after temperature enhancement compared to untreated HeLa cells (Figure S1D).

### Triple dose of irradiation enhances the effect on chlamydial inclusions and their maturation stages

**Figure 6:**
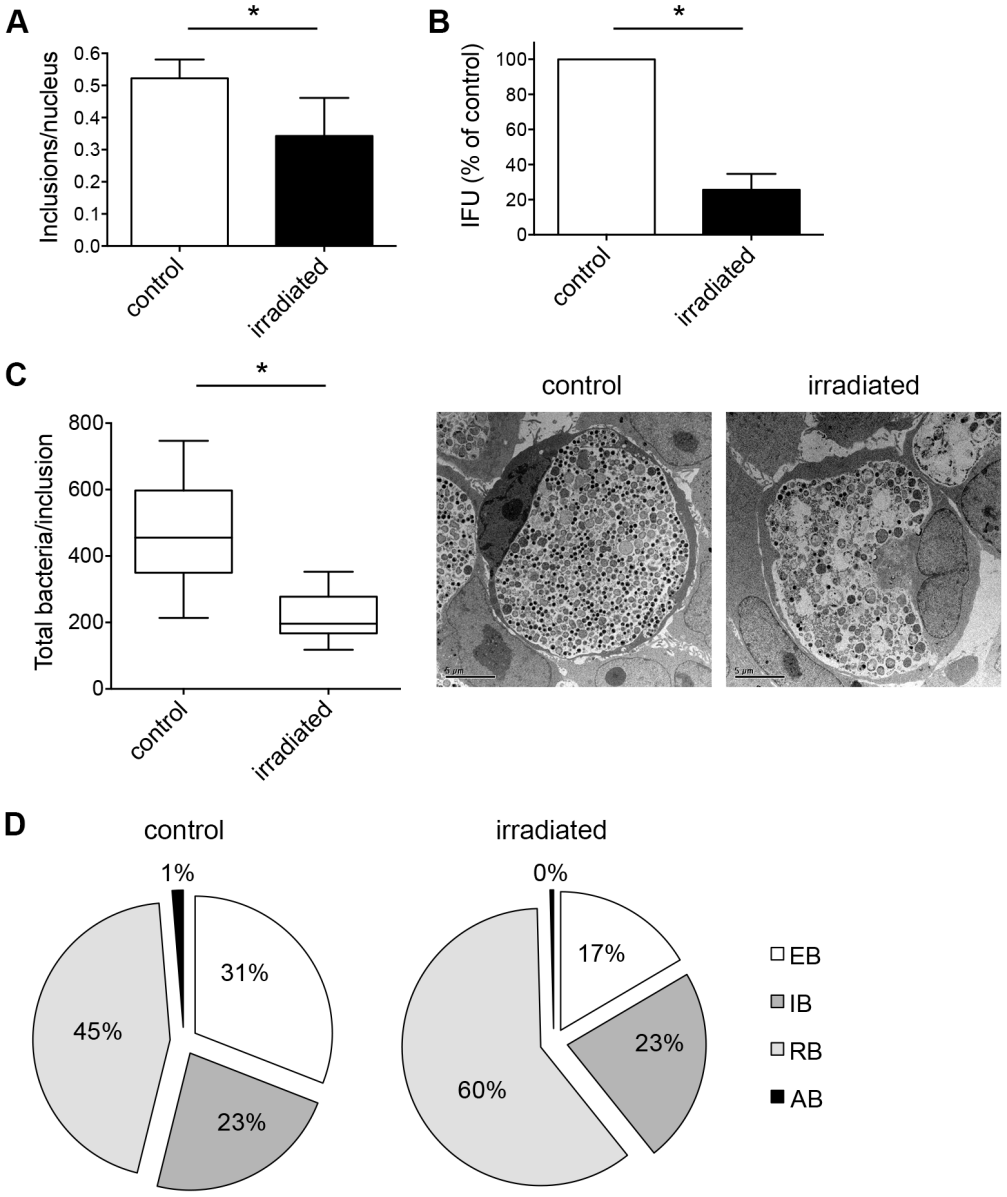
Triple dose of irradiation enhances the effect on chlamydial inclusions and their maturation stages. (A) *C. trachomatis*-infected HeLa cells were irradiated three times at 24, 36 and 40 hpi for each 20 min. Non-irradiated, *C. trachomatis*-infected cells were used as controls. Cultures were fixed 43 hpi and immunolabelled with anti-chlamydial LPS (green) and DAPI (blue). Frequency of inclusions per nucleus was calculated (mean ± SD; * p<0.01; n = 3; *t* test). (B) Infected HeLa cultures were collected and subjected to sub-passage titer analysis. Inclusion forming units (IFU) are presented as percent of control (mean ± SD; * p = 0.0001; n = 3; *t* test). (C) Cultures were fixed 43 hpi and further processed to transmission electron microscopy. The total number of bacteria in ten inclusions per condition was counted: controls (in sum 4627 bacteria) and irradiated inclusions (2179 bacteria). Shown are the means ± SD of chlamydial inclusions (* p<0.0005, n = 10; *t* test). The mid-line shows the median, the box represents the 25^th^ to 75^th^ percentiles whereas the whiskers show the minimum to maximum values. Representative inclusions are shown additionally in the panel. (D) Chlamydial bacteria within ten inclusions (from C) were split according to their morphology into elementary bodies (EB), intermediate bodies (IB) and reticulate bodies (RB). The graphs show the distribution of each maturation stage per condition.

Next, we determined whether multiple doses of irradiation could further reduce the chlamydial inclusion numbers compared to the single irradiation. To test the impact of multiple doses, *C. trachomatis*-infected HeLa cultures were irradiated three times at 24, 36 and 40 hpi for 20 min each at a dose of 3700 W/m^2^ with non-irradiated, *C. trachomatis*-infected HeLa monolayers as controls. Cultures were either collected for sub-passage titer analysis at 43 hpi, or fixed and subsequently immunolabelled according to our general experimental design (Figure 1). The frequency of chlamydial inclusions was reduced in irradiated samples (0.34±0.12 inclusions/nucleus) compared to non-irradiated controls (0.52±0.06 inclusions/nucleus) (Figure 6A). When inclusions were counted directly by immunofluorescence microscopy, the reduction effect of the triple-dose treatment yielded almost identical reduction (34.62%) compared to single-dose irradiation (36.36%). However, a more profound decrease in IFU/mL was observed after threefold irradiation (25.60%±9.14%), compared to that observed after a single dose of irradiation (62.88%±8.41%, Figure 6B)).

Because these data indicate that multiple irradiations have an additive effect on chlamydial infectivity, we examined the ultrastructure of threefold-irradiated chlamydial inclusions. HeLa cells were infected, irradiated three times (24, 36, 40 hpi), fixed after a 3-hour incubation period and further processed for electron microscopy at 43 hpi. We counted the total number of bacteria in irradiated and non-irradiated, *Chlamydia*-infected cells as previously described. Containing 4627 chlamydial bodies in total, non-irradiated inclusions consisted of more than twice as many bacteria as irradiated inclusions (2179 chlamydial organisms, Figure 6C). Similar to single-dose irradiation, chlamydial developmental forms were categorized into different developmental stages (EBs, IBs, RBs, ABs) according to their morphology. Irradiated inclusions contained fewer EBs (16.6%±9.48%) but a higher amount of RBs (60.5%±14.52%) compared to the non-irradiated chlamydial inclusions with 31.3%±13.48% EBs and 45.3%±12.45% RBs, respectively. The distribution of IBs was similar in both conditions with 23.2%±6.85% in non-irradiated and 22.8%±6.91% in irradiated inclusions, respectively. The number of ABs was negligible in both testing conditions (Figures 6D and S3B).

### Irradiation and chlamydial infection trigger an identical subset of released cytokines and chemokines from cells

**Figure 7:**
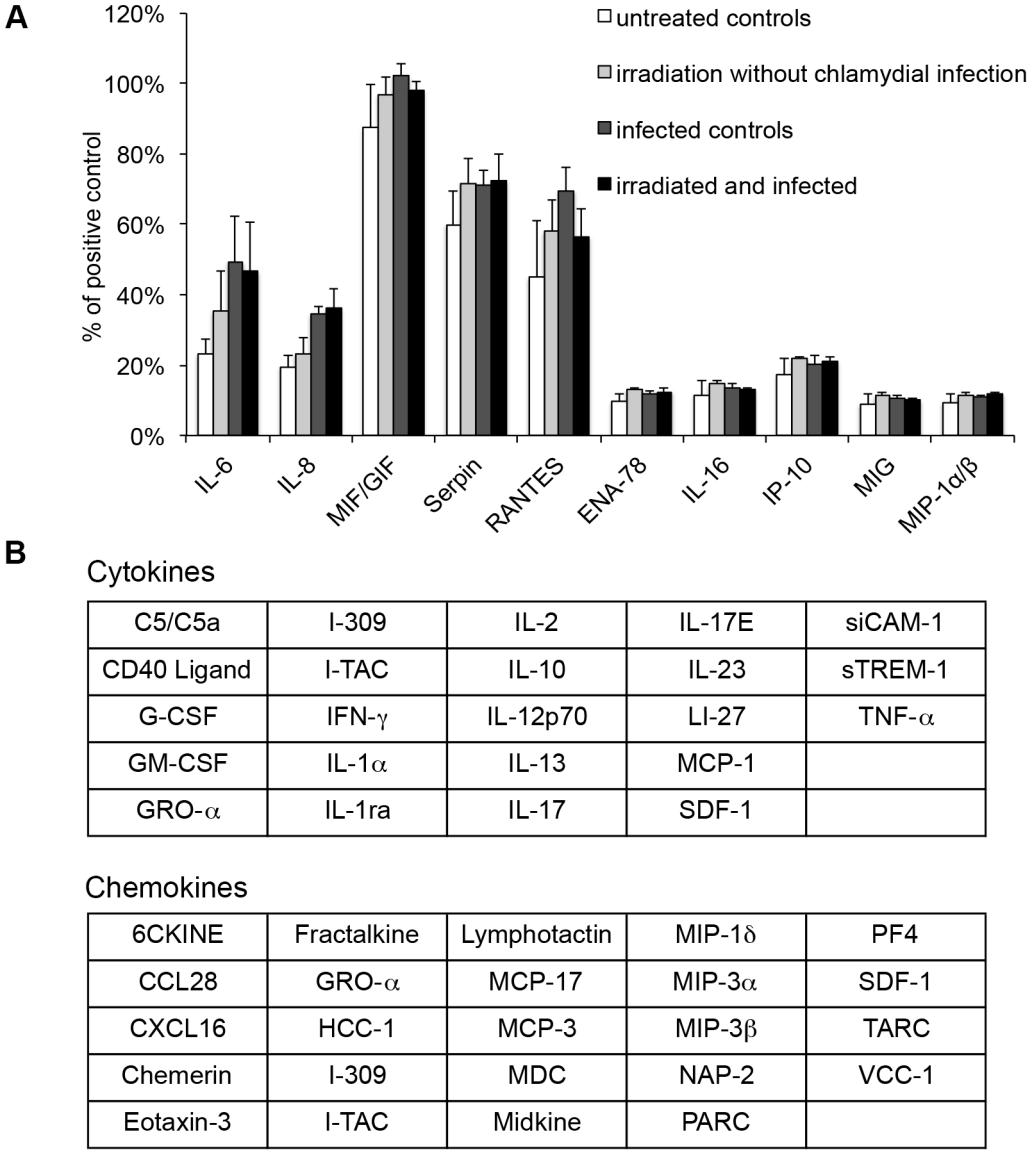
Irradiation and chlamydial infection trigger an identical subset of released cyto- and chemokines from cells. HeLa cells were either infected or not with *C. trachomatis* and irradiated three times (24, 36 and 40 hpi). Supernatant was collected 43 hpi and cytokines as well as chemokines were analyzed using cytokine and chemokine array panel kits. (A) All altered cytokines (IL-6, IL-8, MIF/GIF, RANTES, Serpin E1) and chemokines (ENA-78, IP-10, IL-16, MIG, MIP-1α/β) following (ii) irradiation without chlamydial infection, (iii) infection without irradiation (infected controls), and (iv) irradiation of *Chlamydia*-infected HeLa cells (irradiated and infected) compared to (i) untreated controls are shown. The results are presented as percentage of the positive control (mean ± SEM; n = 3). (B) This table summarizes all tested cytokines and chemokines measured by the assay with no alteration compared to the controls.

Finally, to investigate the response of HeLa cells to irradiation, to chlamydial infection, and to the combination of both, cytokine and chemokine assays were performed. This experiment included four different treatment groups: (i) non-infected HeLa cells without irradiation, (ii) non-infected HeLa monolayers with irradiation, (iii) *C. trachomatis*-infected HeLa cultures without irradiation (infected controls), and (iv) *C. trachomatis*-infected HeLa cultures with irradiation. Triple-dose irradiation (24, 36, 40 hpi) was performed as previously described, the supernatants of each experimental set were collected at 43 hpi, and cytokines and chemokines were analyzed using cytokine and chemokine array panel kits according to manufacturer's instructions. Beforehand, the arrays were validated by assessment of the linearity of internal assay controls determined over time (Figure S2). We observed a similar release pattern in five out of 20 cytokines (IL-6, IL-8, MIF/GIF, RANTES, Serpin E1) and in five out of 24 chemokines (ENA-78, IL-16, IP-10, MIG, MIP-1α/β) in all three treatment groups (ii)-(iv) (Figure 7A). However, expression of most cytokines and chemokines tested did not change under the treatment conditions examined (Figure 7B).

## Discussion

Antimicrobial therapy is the treatment of choice for bacterial infections. However, all pharmacological approaches bear the risk of unwanted side effects such as antibiotic-associated diarrhea in patients treated with amoxicillin-clavulanate, or more severe risks such as cephalosporin-induced (cefotetan) immune hemolytic anemia, drug-induced liver toxicity due to various antimicrobial agents (sulfonamides, macrolides, quinolones) and kidney injury caused by aminoglycosides, vancomycin and others [23].

*Chlamydia trachomatis* is a known agent of ocular and genital infections leading to blindness (Trachoma) and sexually transmitted diseases. Recommended antibiotic therapies to treat these conditions are either by multiple doses of tetracycline or a single dose of azithromycin [10]. However, a recent study has found that the risk of cardiovascular death in patients on azithromycin is elevated, compared to patients treated with amoxicillin or ciprofloxacin [23], [24]. Another well-known factor limiting the efficacy of antimicrobial treatment is the development of antibiotic resistance through acquisition of resistant determinants by horizontal gene transfer and/or other mechanisms [25].

In veterinary medicine, tetracycline-resistant *C. suis* strains carrying the *tet*(C) resistance gene have been described in the USA [26] and Europe [27]–[29]. Very recent findings reported *C. suis* in the eyes of trachoma patients in Nepal [30], highlighting its zoonotic potential. Various studies have detected resistance of *C. trachomatis* to other antibiotics, such as rifampin, *in vitro* [6]–[9]. The obligate intracellular lifestyle of chlamydiae poses additional challenges to antibiotic treatment, as the compound must penetrate the host cell to get access to the membrane-bound intracytoplasmic inclusion.

Facing all these limitations and drawbacks, alternative therapeutic strategies to treat chlamydiae are worth investigating. A small-molecule inhibitor of type III secretion leading to a dose-dependent decrease of chlamydial inclusions in acutely infected host cells was reported by Muschiol *et al*. [31] and other chemical inhibitors of chlamydial development are being explored. Unfortunately, there are few other studies investigating non-chemical agents for inhibition/treatment of chlamydial infections. Ermolaeva *et al*. [32] investigated the effect of non-thermal plasma (NTP) on *C. trachomatis*-infected McCoy cells. NTP is «the flow of partially ionized, neutral gas obtained at atmospheric pressure» with a temperature of 30–40°C. Infected cells were treated with NTP 24 hpi for 2 min and medium was subsequently replaced. Sub-passage titer analysis revealed a reduction by a factor of 1.9×10^6^ compared to untreated and argon-treated controls. Furthermore, the authors could show that treatment of extracellular EBs led to a very low infectivity rate (<0.01%) compared to untreated controls (∼90%). However, there was a decrease of cell viability of approximately 20% at 24 h after treatment. Wasson *et al*. [33] used visible light (violet, 405 nm) to irradiate *C. trachomatis*-infected HeLa cells for 88 seconds (s) using different exposure intensities (5, 10, 20 J/cm^2^) at 2 and 24 hpi, respectively. Using quantitative real-time PCR, they found an inhibitory effect on chlamydial growth in acute and penicillin-induced persistent infections after irradiation. Interestingly, a clinical study found evidence for a lower rate of wound infection following abdominal surgery when patients were subjected to the combination of wIRA (780–1400 nm) and visible light (VIS, 590–780 nm) postoperatively twice a day for 20 min [11]. In the present study, we demonstrate that irradiation of chlamydial EBs with wIRA/VIS prior to infection of cell cultures reduces their infectivity, regardless of the chlamydial strain and the host cell used. We observed a 65% reduction of IFU/mL when irradiated *C. trachomatis* EBs were propagated in HeLa cells and a 90% IFU reduction when irradiated *C. pecorum* EBs were inoculated into Vero cells. Taken together, our findings show that wIRA/VIS-exposure can significantly reduce infectivity of extracellular infectious EBs. Thus, wIRA/VIS irradiation might prove beneficial for inhibiting the transmission of EBs.

We further investigated the effect of single-dose treatment (3700 W/m^2^, 20 min) on fully developed chlamydial inclusions. We investigated two different mammalian cell lines (HeLa, Vero) and two different chlamydial strains (*C. trachomatis*, *C. pecorum*). Our findings demonstrate that a single dose of wIRA/VIS irradiation is sufficient to reduce the amount of chlamydial inclusions *in vitro* regardless of the infection model. Sub-passage titer analysis of irradiated *C. pecorum*-infected Vero cells was performed separately for cells and supernatant. Because we observed similar infectivity reduction in both cell monolayers and supernatants, it seems unlikely that the wIRA/VIS-mediated decrease in inclusion number is due to premature inclusion rupture and subsequent loss of inclusions from the monolayer. Furthermore, we show here an accumulating effect of irradiation by applying multiple wIRA/VIS doses in the course of the infection cycle.

Chlamydiae are not only associated with acute infections but also with a variety of chronic diseases characterized by scarring and inflammation, which result in considerable damage to the host. One example of this process is tubal infertility subsequent to *C. trachomatis*-induced pelvic inflammatory disease. A number of *in vivo* and *in vitro* studies suggest that chlamydiae remain in a non-infectious but viable developmental state (termed either persistence or the chlamydial stress response) during chronic infections [34]. Galasso and Manire [35] demonstrated first that penicillin-exposed *C. psittaci* enters a non-infectious but viable state *in vitro*. Removal of the stressor led to normal progression of the chlamydial development cycle. Since 1961, various stressors responsible for persistence induction *in vitro* have been reported such as amino acid or iron deficiency, IFN-γ exposure, monocyte infection and heat shock [5], [34]. Kahane and Friedman [36] transferred *C. trachomatis*-infected BGM cells to 42°C inducing heat shock. They demonstrated that prolonged exposure to temperatures of 42°C for more than 9 h results in stagnation of the growth cycle and loss of infectivity. Cultures exposed to heat shock for 2 h revealed regular distribution of EBs and RBs whereas cultures treated with 42°C for 5 h predominantly displayed aberrant bodies. In our study, persistence induction and occurrence of ABs was excluded by TEM analysis. No difference in AB frequency was observed between irradiated and non-irradiated cells under any experimental condition tested (i.e. neither single dose in Vero cells infected with *C. pecorum*, nor multiple doses in *C. trachomatis* infected HeLa cells). We could further demonstrate that single dose irradiation reduces the total amount of organisms by about 50% compared to non-irradiated *C. pecorum* inclusions, without altering the distribution of EBs and RBs. Non-irradiated *C. trachomatis* inclusions harbored more than twice as many chlamydiae compared to three times irradiated inclusions. Moreover, three times irradiated *C. trachomatis* inclusions contained fewer EBs and a higher amount of RBs. The absolute number of irradiated RBs was reduced compared to non-irradiated RBs, however. Taken together, we show that irradiation diminished the total amount of chlamydial developmental forms by an unknown mechanism without inducing chlamydial persistence.

The present study investigated not only the effect of wIRA/VIS on chlamydiae, but also examined the host cell response under wIRA/VIS treatment. Thus, the influence of irradiation on two different host cell types was evaluated by cell viability assays as well as Western Blot analysis of cellular stress markers. We demonstrate that host cell viability was not altered by irradiation even at the highest possible dosage of 3700 W/m^2^ and during long-time exposure of 4 h. These findings are in line with a study by Jung *et al*. [15]. They investigated the effect of wIRA irradiation at 2100–2400 W/m^2^ for up to 4 h on human fibroblasts. Recent studies have shown the induction of MMP-1 due to infrared A irradiation [37], [38]. Matrix Metalloproteinase (MMP)-1 contributes to premature skin aging and is expressed as response to ultraviolet radiation (UV) A and UV B [39]. Additionally, Schieke *et al*. [37] reported rapid activation of extracellular signal-regulated kinase 1/2 (ERK 1/2) and p38-mitogen-activated protein kinase (p38) by phosphorylation within 15 min after exposure to infrared A. They further demonstrate that expression of MMP-1 is directly regulated by the ERK 1/2 pathway. In our study, we investigated phosphorylation of ERK 1/2, p38 and Akt in order to examine possible stress response signaling due to wIRA/VIS irradiation. Neither of those stress kinases was activated by irradiation up to 30 min after treatment. Another stress response of cells is the occurrence of autophagy, an organized, endogenous clearance pathway within cells. During autophagy, damaged cell structures are removed by transportation to lysosomes and subsequent degradation [40]. In this study, we included Western blot analysis of LC-3B conversion as a marker of autophagy and detected no signs of autophagy induction in irradiated HeLa cells up to 24 h post treatment. Finally, we assayed cleaved Caspase 7 and 9, as additional sensors of apoptotic cell death. As expected, none of these cell death proteases were activated by wIRA/VIS treatment. Taken together, these data demonstrate that wIRA/VIS does not induce cytotoxicity within host cells at an exposure intensity of 3700 W/m^2^ over the course of 20 min and beyond.

Next, we studied the effect of wIRA/VIS on the host cell cytokine and chemokine response in HeLa cells that were either mock-infected or infected with *C. trachomatis*. In general, bacterial infections induce a pro-inflammatory response within host cells leading to activation of the immune system and subsequent elimination of the pathogens unless the pathogens possess appropriate evasion strategies. Whereas numerous studies have investigated the interaction between host cells and *Chlamydia*, the general importance of pro-inflammatory response to control chlamydial infection is still controversial [41]. Studies investigating the cytokine and chemokine response to chlamydial infection have shown up-regulation of IL-1α, IL-6, IL-11, TGF-β1, GM-CSF, GRO-α and IL-8 in *C. trachomatis*-infected HeLa cells [42]. In our study, we compared the cytokine and chemokine pattern of (ii) non-infected, wIRA-irradiated HeLa cells, (iii) *C. trachomatis*-infected, non-irradiated and (iv) infected, irradiated cultures to (i) non-infected, non-irradiated HeLa cell monolayers. Irradiation was performed three times (24, 36, 40 hpi) and supernatant was collected for subsequent cytokine and chemokine analysis at 43 hpi. Compared to group (i), we observed increased secretion of five cytokines (MIF/GIF, Serpin E1, RANTES, IL-6, IL-8) and five chemokines (ENA-78, IL-16, IP-10, MIG, MIP-1α/β) following wIRA/VIS-exposure, chlamydial infection or both.

We observed an increase of pro-inflammatory IL-6 and IL-8 in *Chlamydia*-infected cells. These results are consistent with previously published observations [19], [21], [43]–[46]. We also detected a thermal-induced increase of IL-6 and IL-8 due to wIRA/VIS irradiation. Jiang *et al*. [47] also reported an up-regulation of IL-6 in mice following challenge with LPS and subsequent elevation of their core temperature to 39.5–40°C. In contrast, Shah *et al*. [48] found no change of IL-6 and IL-8 in HUVEC cells after treating them with an elevated temperature of 40°C for 6 to 12 h.

IL-1α is another pro-inflammatory cytokine strongly interacting with IL-6 and IL-8. Its up-regulation in *Chlamydia*-infected cells has been shown previously in numerous studies [19], [43], [45]. It is released into the supernatant at the end of the chlamydial developmental cycle [45]. The longest incubation period in our setting was 46 hpi and as expected, we did not find an increased secretion of IL-1α.

We also detected an increase of MIF/GIF after chlamydial infection, a pro-inflammatory cytokine promoting the production of tumor necrosis factor (TNF), IFN-γ, IL-1β, IL-2, IL-6 and IL-8 [49]. Törmänkangas *et al*. [21] has reported similar results in *C. pneumoniae*-infected Calu3 cells whereas Johnson [50] found no change of MIF/GIF in murine oviduct cells infected with *C. muridarum* up to 24 hpi.

We found an increased secretion of RANTES in *Chlamydia*-infected cells. Other authors have shown similar data [50], [51], but Buckner *et al*. [19] demonstrated a decrease of RANTES secretion in human polarized endocervical epithelial cells (polA2EN) infected with *C. trachomatis*. Furthermore, wIRA/VIS treatment alone induced RANTES. In contrast, Shah *et al*. [48] found no alteration of RANTES excretion in thermally treated (40°C for 6 to 12 h) primary endothelial cells (HUVEC).

Following chlamydial infection, we further observed a secretion of pro-inflammatory IP-10 (CXCL 10). These results are in line with other authors [21], [50],[51]. In contrast, Buckner *et al*. [19] found a decrease of IP-10 secretion in *C. trachomatis*-infected polA2EN cells. MIG (CXCL 9) is an angiostatic and chemotactic substance closely related to IP-10 [52] and its increase after chlamydial infection was demonstrated in our study as well as in previous publications [50], [51]. Additionally, wIRA/VIS irradiation alone caused a similar secretion of MIG and IP-10 in HeLa cells whereas Shah *et al*. [48] found no change in the secretion of MIG 10 h after treating HUVEC cells with 40°C for 6 to 12 h.

In our study, we observed a release of MIP-1α/β (CCL3/4) into the supernatant after chlamydial infection and/or irradiation. MIP-1α/β is known to be chemotactic for natural killer (NK) cells [42]. Regulation of MIP-1α/β was unaltered by chlamydial infection in murine oviduct cells and McCoy cells [50], [51]. In contrast, up-regulation of MIP-1α/β gene expression has been reported in cervical tissue of mice after infection with *C. muridarum* at 2 and 6 hpi [53]. MIP-1α/β remained unchanged in HUVEC cells when they were incubated at 40°C for 6 and 12 h and measured 10 h after treatment [48].

ENA-78 (CXCL5) is a pro-inflammatory chemokine associated with neutrophil chemotaxis. In a clinical study investigating active trachoma, gene expression of ENA-78 was increased [54]. The authors postulated that ENA-78 might contribute to fibrosis. An increase of ENA-78 gene expression was found at approximately 24 hpi when mice were intra-cervically infected with *C. muridarum* [53].

Serpin E1, also named plasminogen activator inhibitor-1 (PAI-1, SERPINE1), is a known pro-fibrotic factor [55]. To our knowledge, there is no study so far reporting an increase of Serpin E1 due to chlamydial infection. Yang *et al*. [56] stimulated HeLa cells with IL-1β and analyzed the cytokine pattern, reporting no change between the untreated control group and IL-1β-stimulated HeLa cells.

Taken together, we observed a similar pro-inflammatory host cell response in (ii) irradiated but non-infected HeLa monolayers, (iii) non-irradiated, *C. trachomatis*-infected cultures and the combination of both, (iv) irradiated and infected HeLa cells.

Finally, we tried to get insight into the potential mechanism of wIRA/VIS on infected host cells. In a previous study, Hartel *et al*. [11] found a significant increase of subcutaneous oxygen partial pressure and temperature on the skin surface of patients after wIRA/VIS irradiation. Patients underwent abdominal surgery followed by regular postoperative management. Additionally, patients were irradiated twice a day with either visible light (control group) or the combination of both, visible light and wIRA. Subcutaneous temperature was increased from approximately 36°C up to 39°C within 20 min of irradiation compared to the control. These findings support our results regarding the measured temperature increase from 35°C up to approximately 39°C during the wIRA/VIS irradiation (20 min). We mimicked the temperature increase by placing *C. pecorum*-infected Vero cultures in a water bath at 41°C. The results were similar to those following single dose irradiation, indicating that the inhibitory effect of wIRA/VIS on chlamydial inclusions might be explained by the induction of currently unknown thermal effects. According to our experimental setting, these wIRA/VIS-induced thermal effects led to a diminished infectivity of EBs. Furthermore, the effect of a single dose of irradiation on fully developed inclusions (40 hpi) might have been caused by an inhibition of the RB replication or the RB to EB transition. Additional wIRA/VIS treatment at 24 and 36 hpi might support these hypotheses as *C. trachomatis*-infected HeLa cells mainly consist of RBs at 24 hpi and begin to differentiate into EBs at 36 hpi [14].

In summary, we demonstrated that wIRA/VIS irradiation reduces the infectivity of EBs compared to untreated control EBs regardless of the chlamydial strain (*C. pecorum*, *C. trachomatis*). We further show that wIRA/VIS does not induce cytotoxicity in two different cell lines (Vero, HeLa) even at high doses of wIRA/VIS and long-time exposure. Furthermore, we demonstrated that a single dose of irradiation applied at a late stage of the chlamydial developmental cycle (40 hpi) diminished the number of chlamydial bodies within an inclusion and reduced the total amount of inclusions in infected cell cultures. Multiple-dose irradiation (24, 36, 40 hpi) resulted in an even more profound reduction without inducing persistence. Chlamydial infection and/or wIRA/VIS irradiation triggered an identical, pro-inflammatory host cell response as observed by the release of a similar cytokine and chemokine pattern. We suggest further studies are required to fully elucidate the mechanism behind our findings.

## Supporting Information

Figure S1 **Additional Viability Assays.** (A) HeLa cells were irradiated for 20 min (3700 W/m^2^). Cell viability was analyzed using Alamar blue dye at the indicated time points after irradiation (mean ± SD, n = 2) according to the scheme in the upper panel. Untreated controls are set to 100%. (B) HeLa monolayers were irradiated for 1 h and cell viability was determined using Alamar blue at the indicated time points after irradiation as shown in the upper panel. The means ± SD of three determinations within the same experiment are presented. Untreated controls are set to 100%. (C) HeLa cells were irradiated for 4 h and cell viability was analyzed at the indicated time points after irradiation (scheme). The means ± SD of three determinations within the same experiment are shown. (D) HeLa monolayers were placed in a water bath at 41°C for 20 min and the cell viability was determined at the indicated time points. The means ± SD of three determinations within one experiment are illustrated.
(TIF)

Figure S2 **Validation of cytokine and chemokine arrays.** HeLa cells were either infected or not with *C. trachomatis* and irradiated three times (24, 36 and 40 hpi). Supernatant was collected 43 hpi. Cytokines (A) and chemokines (B) were analyzed using cytokine and chemokine array panel kits. The linearity of the internal assay controls determined over time is shown.
(TIF)

Figure S3 **The effect of wIRA/VIS on chlamydial maturation stages.** (A) *C. pecorum*-infected Vero cells were irradiated 40 hpi. Non-irradiated, *C. pecorum*-infected cells are controls. Cultures were fixed 43 hpi with glutaraldehyde, and further processed as described in Material and Methods in the transmission electron microscopy section. Chlamydial bacteria within ten inclusions were split according to their morphology into elementary bodies (EB), intermediate bodies (IB), reticulate bodies (RB) and aberrant bodies (AB). The graph shows the distribution of each maturation stage per condition (mean ± SD; n = 10). (B) *C. trachomatis*-infected HeLa cells were irradiated three times at 24, 36 and 40 hpi for each 20 min. Non-irradiated, *C. trachomatis*-infected cells were used as controls. Cultures were fixed 43 hpi and further processed to transmission electron microscopy. Chlamydial bacteria within ten inclusions were split according to their morphology as described in (A). The graph shows the distribution of each maturation stage per condition (mean ± SD; n = 10).
(TIF)
