# Supplementary figures and images for: Water-Filtered Infrared A Irradiation in Combination with Visible Light Inhibits Acute Chlamydial Infection
Source: PLoS One. 2014 Jul 14;9(7):e102239. doi: 10.1371/journal.pone.0102239 (PMC4096919; doi:10.1371/journal.pone.0102239)

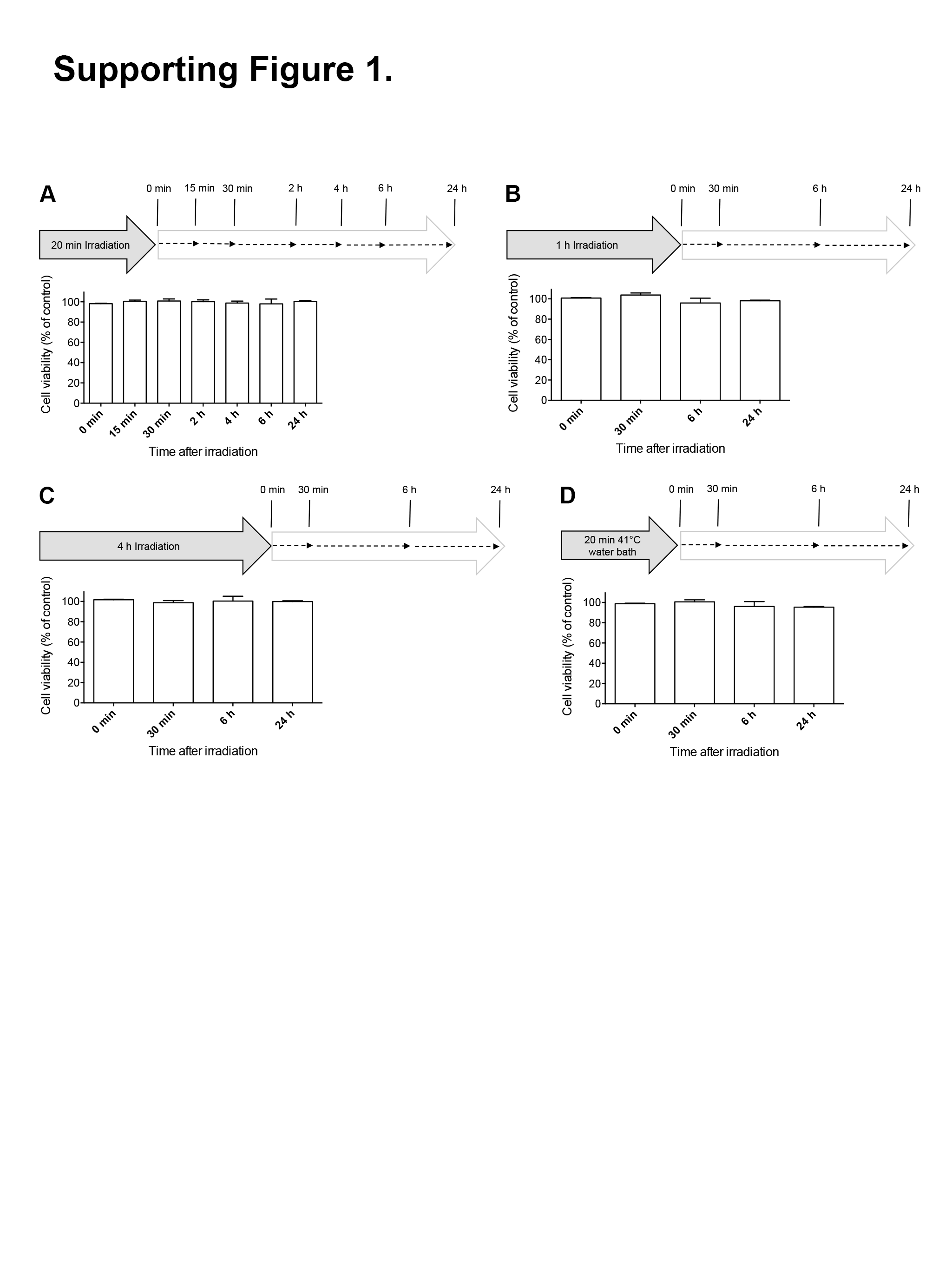

Supplement: Figure S1 — Additional Viability Assays. (A) HeLa cells were irradiated for 20 min (3700 W/m2). Cell viability was analyzed using Alamar blue dye at the indicated time points after irradiation (mean ± SD, n = 2) according to the scheme in the upper panel. Untreated controls are set to 100%. (B) HeLa monolayers were irradiated for 1 h and cell viability was determined using Alamar blue at the indicated time points after irradiation as shown in the upper panel. The means ± SD of three determinations within the same experiment are presented. Untreated controls are set to 100%. (C) HeLa cells were irradiated for 4 h and cell viability was analyzed at the indicated time points after irradiation (scheme). The means ± SD of three determinations within the same experiment are shown. (D) HeLa monolayers were placed in a water bath at 41°C for 20 min and the cell viability was determined at the indicated time points. The means ± SD of three determinations within one experiment are illustrated. (TIF) [file pone.0102239.s001.tif]

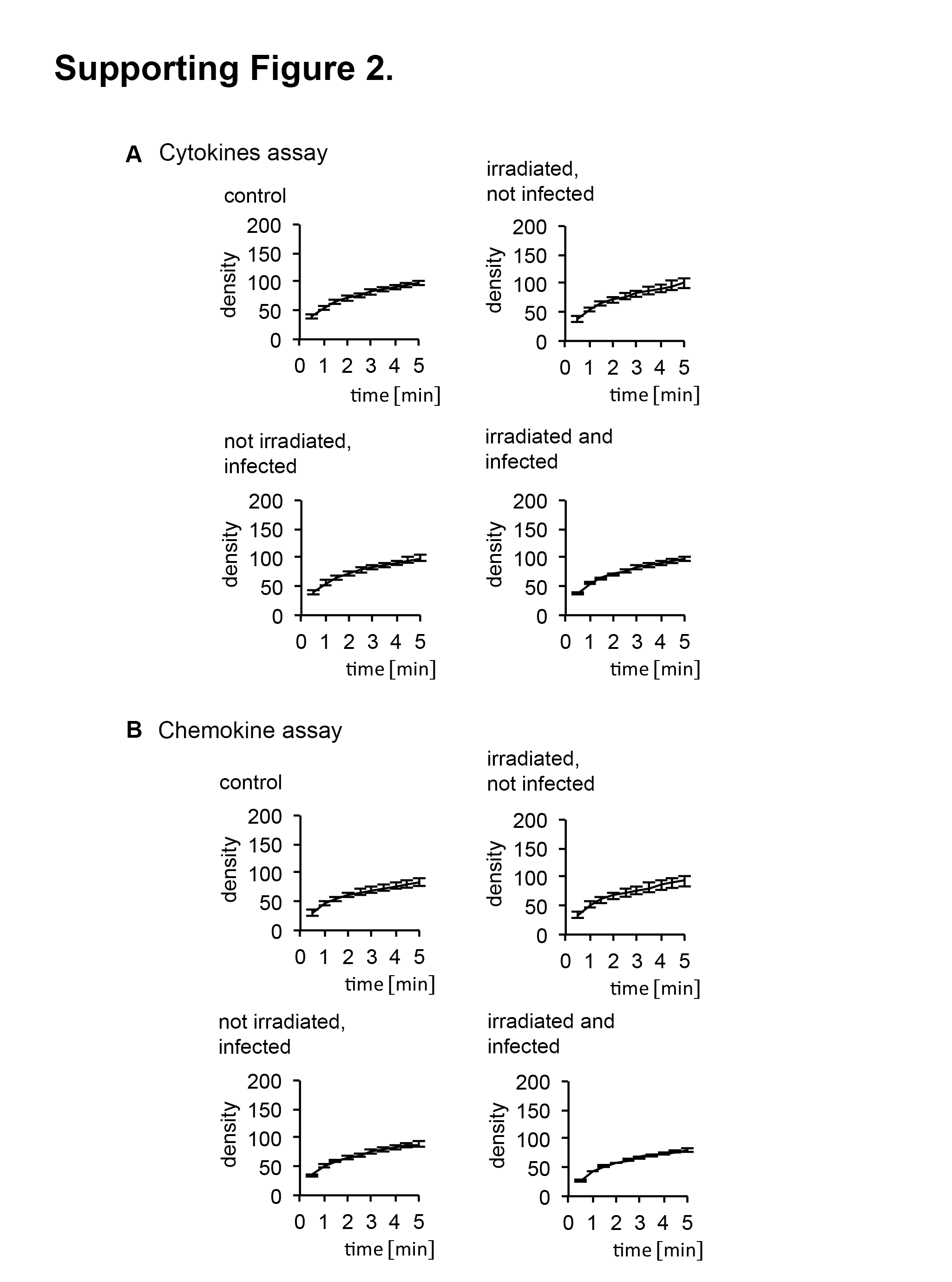

Supplement: Figure S2 — Validation of cytokine and chemokine arrays. HeLa cells were either infected or not with C. trachomatis and irradiated three times (24, 36 and 40 hpi). Supernatant was collected 43 hpi. Cytokines (A) and chemokines (B) were analyzed using cytokine and chemokine array panel kits. The linearity of the internal assay controls determined over time is shown. (TIF) [file pone.0102239.s002.tif]

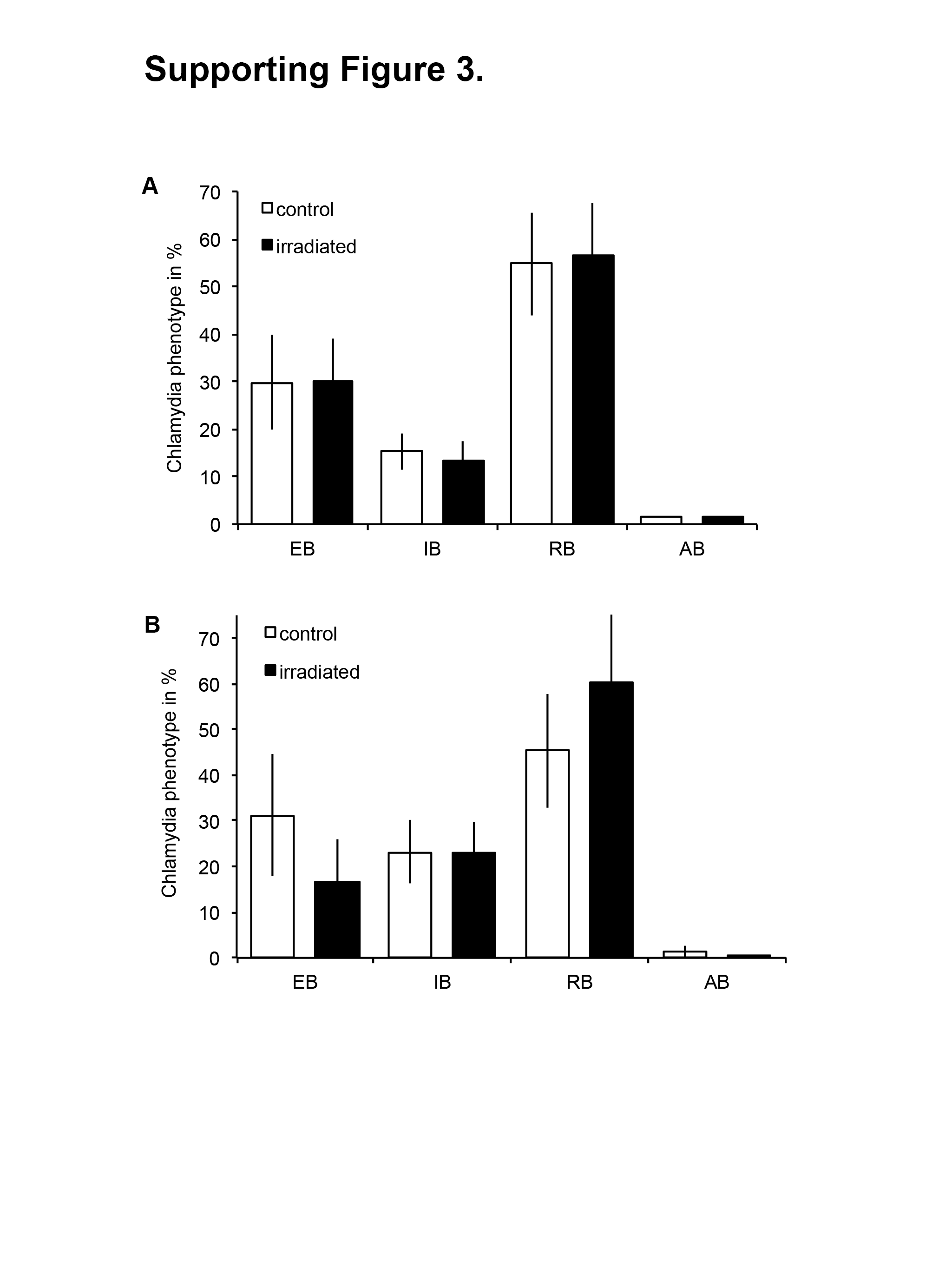

Supplement: Figure S3 — The effect of wIRA/VIS on chlamydial maturation stages. (A) C. pecorum-infected Vero cells were irradiated 40 hpi. Non-irradiated, C. pecorum-infected cells are controls. Cultures were fixed 43 hpi with glutaraldehyde, and further processed as described in Material and Methods in the transmission electron microscopy section. Chlamydial bacteria within ten inclusions were split according to their morphology into elementary bodies (EB), intermediate bodies (IB), reticulate bodies (RB) and aberrant bodies (AB). The graph shows the distribution of each maturation stage per condition (mean ± SD; n = 10). (B) C. trachomatis-infected HeLa cells were irradiated three times at 24, 36 and 40 hpi for each 20 min. Non-irradiated, C. trachomatis-infected cells were used as controls. Cultures were fixed 43 hpi and further processed to transmission electron microscopy. Chlamydial bacteria within ten inclusions were split according to their morphology as described in (A). The graph shows the distribution of each maturation stage per condition (mean ± SD; n = 10). (TIF) [file pone.0102239.s003.tif]
